# The mediating role of psychological capital in health behaviors among elderly nursing home residents

**DOI:** 10.3389/fpsyg.2025.1534124

**Published:** 2025-01-22

**Authors:** Liping Liao, Yunhua Li, Feng Tian, Ju Wu, Jing Zhong, Tingting He, Jinxiu Li

**Affiliations:** ^1^Department of Respiratory and Critical Care Medicine, Zigong First People's Hospital, Zigong, Sichuan, China; ^2^College of Education, Chengdu College of Arts and Sciences, Chengdu, China; ^3^Nursing Department, Zhangjiajie People's Hospital, Zhangjiajie, Hunan, China; ^4^Medical School, Jishou University, Jishou, Hunan, China

**Keywords:** Xiangxi region, nursing homes, elderly, social capital, positive psychological capital, health-promoting behaviors, mediation effect

## Abstract

**Objective:**

This study aims to explore the interactions between social capital, positive psychological capital, and health-promoting behaviors among elderly residents in nursing homes in the Xiangxi region of China.

**Methods:**

A random cluster sampling method was employed to select elderly individuals from 27 nursing homes in the Xiangxi area. Data were collected using the general information questionnaire, the Social Capital Scale, the Positive Psychological Capital Scale, and the Health-Promoting Behaviors Scale. The mediating role of positive psychological capital between social capital and health-promoting behaviors was analyzed.

**Results:**

A total of 341 questionnaires were collected from 27 nursing homes. The data reveals mean scores of 46.83 ± 10.26 for social capital, 72.48 ± 6.39 for positive psychological capital, and 68.25 ± 10.85 for health-promoting behaviors. Mediation analysis shows that the total effect of social capital on health-promoting behaviors was 0.800 (95% CI: 0.726, 0.873), with a direct effect of 0.478 (95% CI: 0.379, 0.577), accounting for 59.75% of the total effect. The indirect effect, mediated by positive psychological capital, was 0.321 (95% CI: 0.233, 0.409), contributing to 40.13% of the total effect.

**Conclusion:**

Positive psychological capital acts as a mediating variable between social capital and health-promoting behaviors. Future interventions designed to enhance health-promoting behaviors must consider both social and psychological capitals to fully leverage their interplay and further promote healthy aging.

## 1 Introduction

The trend of global aging is intensifying. According to predictions by the World Health Organization (WHO), the global population aged 60 and over is expected to increase from 1 billion in 2020 to 1.4 billion by 2030 (Rui, 2023). By 2050, this number is projected to double, reaching 2.1 billion (Rui, 2023). As the most populous country in the world, China has also fully entered an aging society (Hong et al., 2023). Based on the latest statistics, the population aged 60 and above now accounts for 19.8% of the total population in China (NBS, 2022). Furthermore, this proportion is expected to continue rising in the coming years. These demographic trends pose significant challenges for public health policies and social service systems, particularly in promoting the health and welfare of the elderly.

As individuals age, there is an inevitable decline in the function of various systems and organs, which naturally increases the risk of chronic diseases and can lead to significant deterioration in overall health (Liberale et al., 2020; Ou et al., 2022; Rohrmann, 2020). Specifically, the elderly population is commonly afflicted with chronic conditions such as cardiovascular diseases, diabetes, and osteoporosis (Kardas et al., 2021; Kim, 2021; Liberale et al., 2020). These illnesses not only directly impact their daily functional abilities but also significantly reduce their quality of life (Pretto et al., 2020; Tinoco et al., 2021). Moreover, the management and treatment of these health issues not only place a financial burden on individuals and their families but also exert pressure on the entire healthcare system (Giovanni et al., 2020; Zare et al., 2020). Crucially, elderly individuals residing in nursing homes represent a group with a high incidence of chronic diseases, highlighting the need for focused attention on this population (Zhu et al., 2023). However, in most nursing homes in China, while basic medical and living support can be provided, there is often insufficient attention to promoting the overall well-being of the elderly—including physical, psychological, and social dimensions (Gordon et al., 2020; Wang et al., 2018). Existing research indicates that healthy aging requires more than just the treatment of diseases; it is crucial to adopt preventive measures, including enhancing the social and positive psychological capital of the elderly to support their health-promoting behaviors (Xu and Zhao, 2022; Afrashteh et al., 2024; Ye et al., 2024; Jeste et al., 2022). In this context, the impact of social capital and positive psychological capital on elderly health behaviors is particularly significant (Xu and Zhao, 2022; Afrashteh et al., 2024). Social capital can enhance social networks and community participation, providing the necessary support systems that help the elderly access health information and resources, thereby facilitating positive health behaviors (Xu and Zhao, 2022; Afrashteh et al., 2024). Additionally, positive psychological capital, characterized by hope, resilience, optimism, and self-efficacy, can motivate elderly individuals to face health challenges, maintaining a positive outlook and active lifestyle (Xu et al., 2024).

In the context of healthy aging, the concept of social capital has increasingly been incorporated into strategies for health promotion and disease prevention (Lu et al., 2023). Social capital is typically defined as the resources an individual can mobilize through their social networks, which not only facilitate personal actions but also form part of the social structure (Lu et al., 2023; Xu, 2018). Individual social capital is manifested in six dimensions: social participation, social connections, social support, reciprocity, trust, and a sense of belonging (Lu et al., 2023; Xu, 2018). Specifically, social participation refers to the frequency and extent to which elderly individuals engage in various activities and organizations within and outside nursing homes or communities (Jiang and Liu, 2023; Yuying and Jing, 2022). Social connections refer to the frequency and quality of interactions that elderly individuals have with family, friends, and other people both inside and outside the institution (Sullivan et al., 2021; Htun et al., 2024). Social support refers to the types and extent of assistance that elderly individuals can receive from their social networks when they need help, including emotional support, informational support, and material aid (Paudel and Tiwari, 2022; Liu et al., 2023). Reciprocity involves mutual assistance behaviors between elderly individuals and others. Trust refers to the level of trust that elderly individuals have in those around them, such as family members, friends, neighbors, and caregivers, as well as their trust in social institutions like the healthcare system and government agencies (Chen and Zhu, 2021; Shie et al., 2022). A sense of belonging describes the elderly individuals' feelings of affiliation and identity with their community or nursing home, which can enhance their social identity and life satisfaction (Chu et al., 2022; Park et al., 2020).

Extensive research has robustly confirmed that social capital and its various dimensions play a crucial role in the psychological and physiological health of the elderly, effectively preventing chronic diseases and reducing mortality rates (Gontijo et al., 2019; Liang et al., 2020). The mechanisms through which social capital promotes health behaviors can be specifically summarized as follows: First, robust social networks provide crucial information about healthy lifestyles, encouraging individuals to adopt habits such as healthy eating and regular exercise (Hong et al., 2018; Alexandre et al., 2021). Second, social support reduces psychological stress and feelings of loneliness, maintains mental health, and encourages active social participation, which is especially important for the elderly (Choi et al., 2021; Hosseingholizadeh et al., 2019; Dakua et al., 2023; Mieziene et al., 2022). Lastly, reciprocal social behaviors increase the social rewards of participating in group activities, such as joining community health programs or group sports, thereby motivating continued engagement in these activities (Brennan-Ing et al., 2023; Emmering et al., 2018). Through these mechanisms, social capital not only supports the cultivation of individual health behaviors but also directly enhances the quality of life related to health. However, despite current research revealing a positive correlation between social capital and health behaviors, the deeper mechanisms of action require further empirical research, particularly in how enhancing social capital in nursing homes can promote elderly health behaviors and psychological well-being.

Psychological capital refers to the positive psychological state and resources an individual exhibits when facing challenges and stress (Shi, 2013; Youssef-Morgan and Luthans, 2015). This concept, introduced by Luthans and others, is designed to measure and enhance an individual's adaptability and efficacy in complex environments (Shi, 2013; Youssef-Morgan and Luthans, 2015). In the elderly population, psychological capital is particularly significant (Jurek and Niewiadomska, 2021; Leonti and Turliuc, 2024). Extensive research has confirmed its close association with psychological health and quality of life in this group. Psychological capital comprises four core dimensions: self-efficacy, hope, resilience, and optimism (Shi, 2013; Youssef-Morgan and Luthans, 2015). Self-efficacy refers to an individual's confidence in their ability to accomplish specific tasks (Yu et al., 2023; Kim et al., 2020). Hope refers to a positive sense of expectation and the motivation to achieve goals successfully (Sand and Bristle, 2024). Resilience refers to the ability to recover and adapt in the face of adversity (Lavretsky, 2021). Optimism is defined as the tendency to hold positive expectations for the future (Yue et al., 2022).

Research indicates that psychological capital plays a pivotal role in promoting the health and well-being of the elderly (Ahmadboukani et al., 2023). Specifically, psychological capital interacts with the health behaviors and physiological health of the elderly through multiple mechanisms (Ahmadboukani et al., 2023). Firstly, psychological capital enhances the psychological adaptability of the elderly, allowing them to maintain better emotional balance and mental states when facing chronic illnesses or life stressors (Sadeghi and Bavazin, 2019; Li et al., 2022). Additionally, a high level of psychological capital encourages elderly individuals to engage more actively in health-promoting behaviors, such as regular physical activity, balanced diets, and social interactions, which in turn improve their physiological health and psychological well-being (Yoo, 2020). In the context of nursing home environments, strengthening the psychological capital of the elderly is particularly important as these settings can often provoke feelings of loneliness and neglect (Afrashteh et al., 2024). Strategically enhancing the self-efficacy, hope, resilience, and optimism of elderly residents can not only improve their daily functioning and quality of life but also facilitate the broader goal of achieving healthy aging.

Health-promoting behaviors refer to any active actions taken by an individual to enhance, protect, or maintain their health (Xie et al., 2022). These behaviors encompass a wide range, including regular physical activity, a balanced diet, adequate sleep, conscious stress management, avoidance of harmful substances, and regular health check-ups (Xie et al., 2022). Health-promoting behaviors are not only concerned with disease prevention but also involve actively improving an individual's overall well-being and quality of life (Xie et al., 2022; Spring et al., 2019). For instance, Mo et al. found that individuals engaging in more health-promoting behaviors often experience higher quality of life and greater feelings of happiness (Mo et al., 2022). Therefore, in the elderly population, especially those residing in nursing homes, exploring the underlying mechanisms that form their health behaviors is crucial. This not only helps improve their quality of life but also provides a scientific basis for formulating effective health promotion strategies to facilitate the achievement of healthy aging.

In summary, while existing research has explored the individual effects of social capital and positive psychological capital on elderly health, few studies have examined how these two factors simultaneously influence health-promoting behaviors, particularly in nursing home residents. Furthermore, the potential mediating role of positive psychological capital in the relationship between social capital and health behaviors remains underexplored. This study aims to address these gaps by investigating the complex relationships among social capital, positive psychological capital, and health-promoting behaviors. Specifically, we analyze the mediating effect of positive psychological capital between social capital and health-promoting behaviors, and propose a mediation model to better understand how these factors interact. By doing so, this research seeks to provide empirical insights that could inform interventions aimed at promoting positive health behaviors among elderly residents in nursing homes, ultimately contributing to the development of effective strategies for healthy aging in this population.

This study uses Nola J. Pender's Health Promotion Model to explore the relationships between social capital, positive psychological capital, and health-promoting behaviors (Cardoso et al., 2021). Introduced in 1982, the model focuses on how individual health behaviors are influenced by factors such as personal history, biopsychosocial characteristics, perceived barriers and benefits, self-efficacy, and situational influences. It is widely used in elderly health research to understand the factors affecting health behaviors among older adults. To further clarify the relationships among the three variables, this study proposes the following hypotheses:

Hypothesis 1: There is a positive correlation between social capital and positive psychological capital.
Hypothesis 2: There is a positive correlation between social capital and health-promoting behaviors.
Hypothesis 3: There is a positive correlation between positive psychological capital and health-promoting behaviors.
Hypothesis 4: Positive psychological capital mediates the relationship between social capital and health-promoting behaviors.

## 2 Materials and methods

### 2.1 Study participants

**Figure 1:**
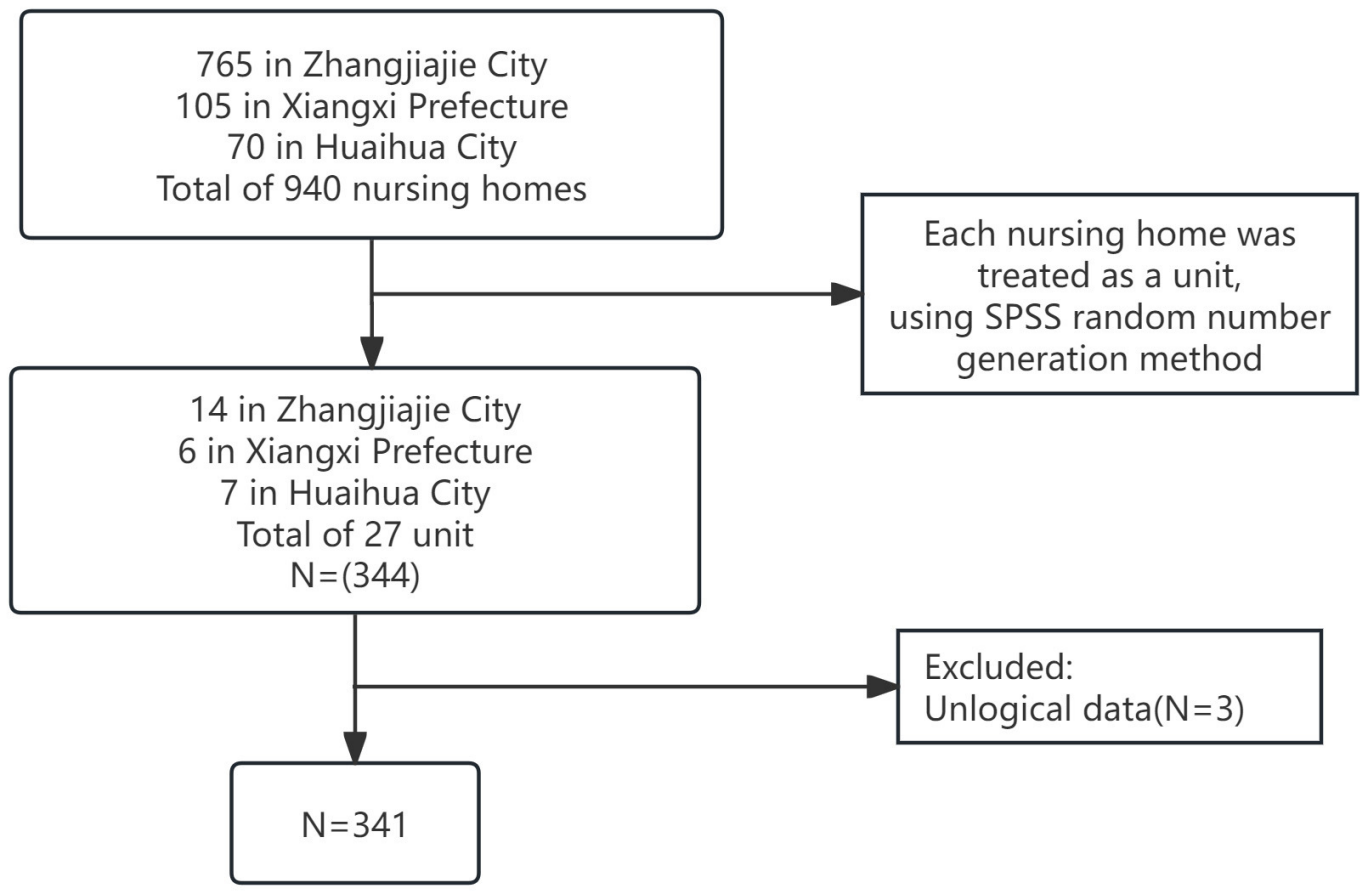
Flow diagram of inclusion criteria and exclusion criteria.

The study was conducted with elderly residents from nursing homes in the Xiangxi area, collected from June 2023 to April 2024. Due to the multivariate nature of the study, the sample size was set to 5–10 times the number of independent variables (Harrell, 2001). The study included six variables from general sociodemographic data, six dimensions of social capital, four dimensions of positive psychological capital, and six dimensions of health-promoting behaviors, totaling 22 independent variables. Calculations determined that the required sample size ranged from 110 to 220 participants, taking into account a 20% margin for invalid questionnaires, which necessitated distributing between 132 and 264 questionnaires. Additionally, considering the minimum sample size of 150 required for equation modeling, with an optimal sample size of over 200, the final sample included 341 participants. A random cluster sampling method was used, covering 940 nursing homes in the region including 765 in Zhangjiajie City, 105 in Xiangxi Prefecture, and 70 in Huaihua City. Each nursing home was treated as a unit; using the SPSS random number generation method, 27 units were randomly selected, from which elderly individuals meeting the inclusion criteria were chosen as the study participants (Figure 1).

Inclusion criteria for this study are as follows: (1) Age and nationality requirements: Participants must be Chinese citizens aged 60 years and older, residing in nursing homes. (2) Cognitive state: Participants must have a clear state of consciousness without any cognitive impairments, ensuring they can understand the questionnaire content and respond accurately. (3) Cooperation ability: Participants should be able to actively cooperate in the survey, including understanding questions and responding appropriately. (4) Self-care ability: Participants must possess basic daily living self-care skills. (5) Informed consent: Individuals must be capable of signing a consent form.

Exclusion criteria include: (1) Mental and cognitive health: Individuals with psychiatric disorders (such as depression or schizophrenia), dementia, or severe cognitive impairments are excluded. (2) Communication abilities: Individuals with communication barriers due to neurological diseases, hearing impairments, or speech disorders that prevent clear expression are also excluded.

### 2.2 Survey methodology

The data collection for this study was conducted with significant support and assistance from the Elderly Care Department of the Civil Affairs Bureau in the surveyed region. At the beginning of the research, we obtained a comprehensive list and basic information about the nursing institutions in the participating area from the Civil Affairs Bureau. After obtaining explicit consent from the directors of the nursing homes and the elderly residents, we arranged on-site, face-to-face surveys. The survey was conducted using the “Questionnaire Star” platform, a widely used online tool for data collection and management in China.

To minimize self-report bias, several measures were implemented throughout the survey process. Research team members carefully explained the purpose and content of the study to the elderly participants, ensuring that they fully understood the significance of their participation. In addition, informed consent was obtained from each participant, with particular attention paid to ensuring that they felt comfortable and free from any social pressure or expectations that might influence their responses. Furthermore, to further reduce potential social desirability bias, the research team read the survey items aloud and recorded the participants' responses verbatim. This approach ensured that the participants' answers accurately reflected their true opinions, rather than being influenced by a desire to please the interviewer or conform to social norms. Finally, to maintain the integrity of the data, researchers checked the accuracy and completeness of the questionnaires in real-time, verifying any errors or omissions immediately with the participants to ensure the collected data were accurate and complete.

### 2.3 Research instruments

#### 2.3.1 General information

The general information includes gender, age, ethnicity, marital status, educational level, and personal monthly income.

#### 2.3.2 Social capital scale

This scale, developed by Xu et al., consists of 22 items across six dimensions: social participation, social connections, social support, reciprocity, trust, and sense of belonging (Xu, 2018). The frequency of participation is rated on a scale from “never” to “often,” with “never” scoring 1 point, “seldom” 2 points, “sometimes” 3 points, “often” 4 points, and “always” 5 points. Higher scores in each dimension indicate a higher level of social capital. The scale was developed by Chinese scholars specifically for the elderly population, demonstrating strong cultural relevance and adaptation. The overall Cronbach's alpha coefficient for the scale is 0.919, with individual dimension alphas ranging from 0.652 to 0.940. In this study, the Cronbach's alpha for the scale was 0.91, indicating good internal consistency and suitability for the research purposes.

#### 2.3.3 Positive psychological capital scale

This study used the Elderly Psychological Capital Scale developed for Older Adults developed by Shi Hui, a localized measurement tool specifically designed to assess the positive psychological states of Chinese older adults with good cultural adaptability (Shi, 2013). The scale comprises 20 items across four dimensions: self-efficacy, integrity and stability, resilience, and gratitude and dedication. It employs a 5-point Likert scale for scoring, with higher scores indicating a higher level of psychological capital among the elderly. The overall Cronbach's alpha coefficient of the scale is 0.876, with dimension-specific Cronbach's alpha coefficients ranging from 0.609 to 0.789, indicating good reliability and validity. In this study, the scale's Cronbach's alpha coefficient is 0.89, indicating strong internal consistency.

#### 2.3.4 Health-promoting lifestyle profile II revised scale

In 2016, Wen and colleagues translated and revised the HPLP-II into a 40-item revised version that was tailored for the Chinese older adult population and is widely used as a tool to measure health-promoting behaviors in Chinese older individuals (Wenjun et al., 2016). The scale includes 40 items across six dimensions: interpersonal relations, nutrition, health responsibility, physical activity, stress management, and spiritual growth. It uses a 4-point Likert scale for scoring, with total scores ranging from 40 to 160: scores from 40 to 69 indicate poor health-promoting behavior, 70 to 99 are average, 100 to 129 are good, and 130 to 160 are excellent. The overall Cronbach's alpha coefficient of the scale is 0.920, with split-half reliability ranging from 0.64 to 0.78 and dimension-specific Cronbach's alpha coefficients ranging from 0.63 to 0.81. The test-retest reliability of the scale is 0.69, and in this study, the Cronbach's alpha is 0.93.

### 2.4 Ethical principles

This study was reviewed and approved by the Biomedical Ethics Committee of Jishou University, under the code JSDX-2024-0072. Prior to data collection, the purpose and content of the study were explained to the elderly participants, and they were informed that they could withdraw from the study at any time.

### 2.5 Statistical analysis

**Figure 2:**
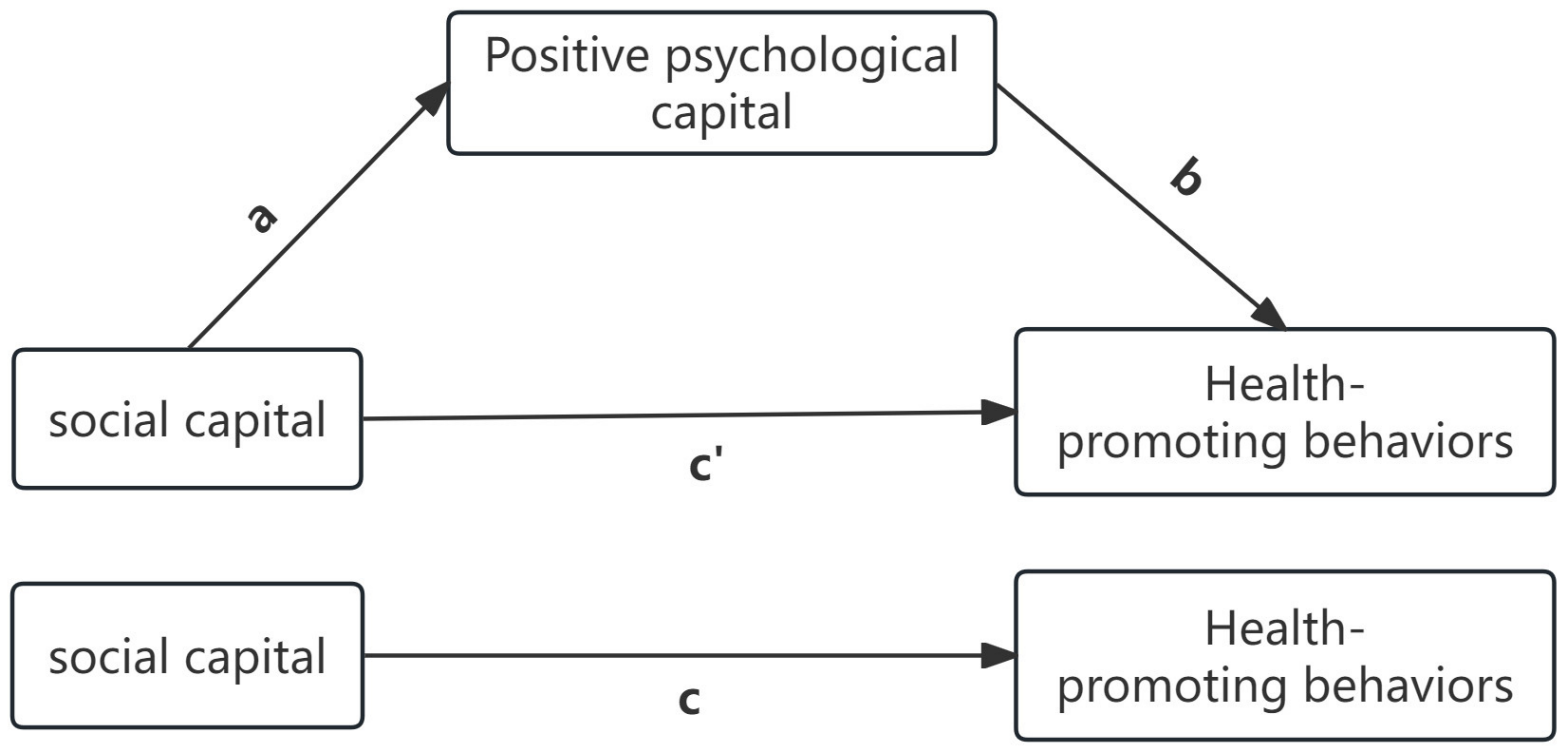
Conceptual mediation model of social capital, positive psychological capital, and health promoting behaviors.

Statistical analysis was conducted using SPSS version 25.0. Descriptive statistics were reported as percentages for categorical data and means ± standard deviations for continuous data. Differences in total scores of social capital, positive psychological capital, and health-promoting behaviors across different sociodemographic groups were analyzed using *t*-tests and one-way ANOVA. The relationships among social capital, positive psychological capital, and health-promoting behaviors were examined using two separate multivariate linear regression models. Mediation analysis was performed using the SPSS PROCESS plugin developed by Hayes, with social capital as the predictor variable, scores of positive psychological capital as the mediator, and total scores of health-promoting behaviors as the outcome variable. A bootstrapping procedure with 5,000 samples was used to test the estimated mediation effect (a^*^b). The theoretical model assessing the mediating role of positive psychological capital in the relationship between social capital and health-promoting behaviors is outlined as follows: “c′” represents the direct effect of social capital on health-promoting behaviors, the product of “a” and “b” (ab) represents the indirect effect of social capital on health-promoting behaviors through positive psychological capital, and “c” represents the total effect of social capital on health-promoting behaviors, which is the sum of “c′” and ab (Figure 2). In all analyses, sociodemographic characteristics (gender, age, ethnicity, marital status, educational level, and personal monthly income) were included as covariates to control for potential confounding effects.

## 3 Results

### 3.1 Sociodemographic characteristics

In this study, 344 questionnaires were initially distributed. After excluding questionnaires with illogical responses, 341 valid questionnaires were recovered, resulting in an effective response rate of 99.13%. The sample included 341 elderly individuals with an average age of 76.13 ± 9.58, ranging from 60 to 101 years. The sample comprised 234 males (68.62%) and 107 females (31.38%); 149 participants were Han Chinese (43.7%), and 192 were from minority ethnic groups (56.3%). Among the participants, 26 had spouses (7.62%), and 315 were without spouses (92.38%). Regarding educational levels, 278 had elementary school education or lower (81.52%), 30 had junior high school education (8.8%), 17 had high school or vocational school education (4.99%), 12 had some college education (3.52%), and 4 had a bachelor's degree or higher (1.17%). Personal monthly income levels were as follows: ≤ 1,000 RMB for 265 participants (77.71%), 1,001–3,000 RMB for 21 participants (6.16%), 3,001–5,000 RMB for 37 participants (10.85%), and ≥5,000 RMB for 18 participants (5.28%). Table 1 presents the sociodemographic characteristics of the participants.

**Table 1:** Different socio-demographic characteristics of social capital, positive psychological capital, and health promotion behaviors (*n* = 341).

	***N* (%)**	**Social capital**	***P***	**Positive psychological capital**	***P***	**Health-promoting behaviors**	***P***
Totals		46.83 ± 10.26		72.48 ± 6.39		68.25 ± 10.85	
Gender			**< 0.001**		**< 0.001**		**< 0.001**
Male	234 (68.62)	44.23 ± 9.25		71.32 ± 5.84		66.15 ± 9.66	
Female	107 (31.38)	52.52 ± 10.10		75.01 ± 6.83		72.83 ± 11.89	
Age (years)			**< 0.001**		**< 0.001**		**< 0.001**
60–69	93 (27.27)	41.97 ± 7.53		69.96 ± 4.84		64.39 ± 6.77	
70–79	110 (32.26)	44.65 ± 8.92		71.33 ± 5.01		66.73 ± 9.07	
≥80	138 (40.47)	51.85 ± 10.71		75.10 ± 7.30		72.07 ± 13.01	
Nation			**< 0.001**		**< 0.001**		**< 0.001**
Han ethnic group	149 (43.70)	49.48 ± 10.35		74.58 ± 6.62		71.68 ± 11.62	
National minority	192 (56.30)	44.77 ± 9.73		70.85 ± 5.71		65.58 ± 9.41	
Marital status			**0.013**		0.59		**0.039**
Married	26 (7.62)	51.62 ± 10.88		73.46 ± 9.80		72.46 ± 12.20	
Unmarried	315 (92.38)	46.43 ± 10.13		72.40 ± 6.04		67.90 ± 10.68	
Education level			**< 0.001**		**< 0.001**		**< 0.001**
Primary school or below	278 (81.52)	45.14 ± 8.88		71.38 ± 5.23		66.38 ± 8.87	
Junior high school	30 (8.80)	49.80 ± 13.81		73.93 ± 7.64		70.00 ± 12.16	
High school	17 (4.99)	56.59 ± 9.87		79.06 ± 8.50		79.41 ± 13.57	
Junior college	12 (3.52)	57.83 ± 6.41		80.00 ± 6.95		82.00 ± 6.25	
University and above	4 (1.17)	67.50 ± 13.89		87.25 ± 9.07		96.00 ± 26.01	
Personal monthly income (RMB)			**< 0.001**		**< 0.001**		**< 0.001**
≤ 1,000	265 (77.71)	43.95 ± 8.06		70.98 ± 4.78		65.37 ± 7.92	
1,001–3,000	21 (6.16)	56.90 ± 13.67		76.00 ± 8.89		77.43 ± 15.11	
3,001–5,000	37 (10.85)	54.57 ± 8.07		76.08 ± 6.67		74.78 ± 8.83	
≥5,000	18 (5.28)	61.56 ± 11.19		83.06 ± 8.79		86.56 ± 16.30	

Bold values represent statistically significant results (*p* < 0.05).

### 3.2 Sociodemographic characteristics and their impact on social capital, positive

Psychological Capital, and Health-Promoting Behaviors Statistical significance was found in the associations of social capital and health-promoting behaviors with sociodemographic characteristics such as gender, age, ethnicity, marital status, educational level, and personal monthly income (Table 1). However, for positive psychological capital, all characteristics except marital status showed statistical significance.

### 3.3 Relationships between social capital, positive psychological capital, and health-promoting behaviors

The results of the multivariate linear regression models examining the relationships between social capital, positive psychological capital, and health-promoting behaviors are as follows: After controlling for gender, age, ethnicity, marital status, educational level, and personal monthly income, Model 1 shows that social capital (*B* = 0.395, *P* < 0.001) is significantly related to positive psychological capital. Model 2 indicates that both social capital (*B* = 0.441, *P* < 0.001) and positive psychological capital (*B* = 0.627, *P* < 0.001) are related to health-promoting behaviors (Table 2).

**Table 2:** Multiple linear regression of the relationship between social capital, positive psychological capital, and health-promoting behaviors (*n* = 341).

	**Positive psychological capital**				**Health-promoting behaviors**			
	**Unst**		**St**	***P***	**Unst**		**St**	***P***
	***B***	**SE**	**Beta**		***B***	**SE**	**Beta**	
(Constant)	49.771	2.417		**< 0.001**	2.576	5.541		0.642
Gender	−0.345	0.551	−0.025	0.531	0.514	0.838	0.022	0.540
Age (years)	0.369	0.320	0.047	0.250	−0.678	0.488	−0.051	0.166
Nation	−1.229	0.486	−0.096	**0.012**	−0.876	0.746	−0.040	0.241
Marital status	1.993	0.894	0.083	**0.026**	−0.512	1.369	−0.013	0.709
Education level	1.044	0.344	0.134	**0.003**	1.051	0.531	0.080	**0.049**
Average monthly family income	0.385	0.362	0.053	0.288	0.872	0.552	0.071	0.115
Social capital	0.395	0.028	0.635	**< 0.001**	0.441	0.054	0.417	**< 0.001**
Positive psychological capital					0.627	0.083	0.369	**< 0.001**
	*R*^2^ = 0.575; *F* = 66.791; *P* < 0.001				*R*^2^ = 0.659; *F* = 83.286; *P* < 0.001			

Bold values represent statistically significant results (*p* < 0.05).

### 3.4 Mediating effect of positive psychological capital between social capital and health-promoting

**Figure 3:**
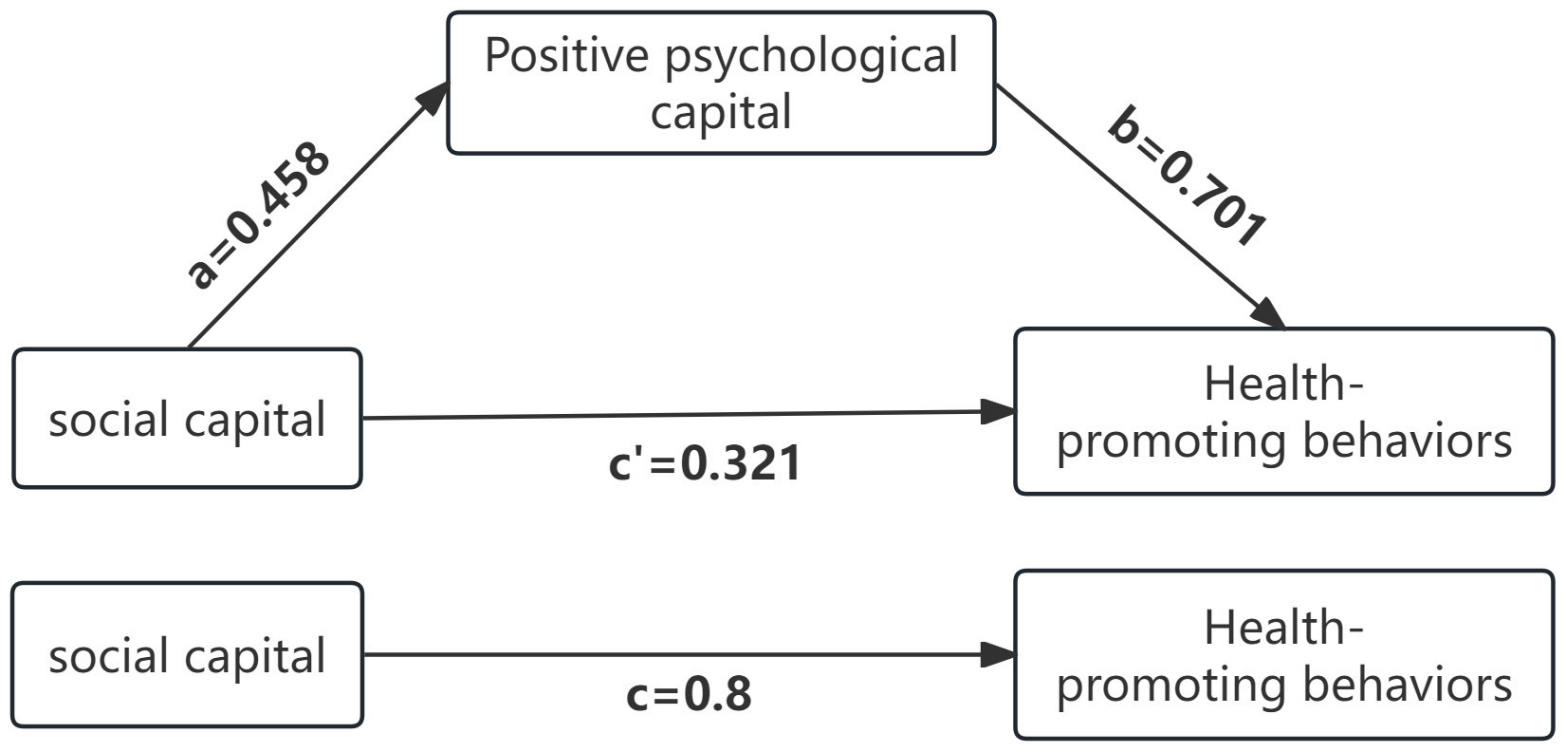
The mediating effect of positive psychological capital between social capital and health promotion behavior.

The mediation analysis shows that positive psychological capital significantly mediates this relationship. Specifically, the indirect effect of positive psychological capital (path a^*^b) was found to be 0.321, with a 95% confidence interval (CI) ranging from 0.233 to 0.409, indicating that the mediating effect is statistically significant as the confidence interval does not include zero (Table 3 and Figure 3).

**Table 3:** Mediating effects of positive psychological capital between social capital and health promotion behaviors (*n* = 341).

	***B***	**95% CI**	**SE**	***P***	**Percentage (%)**
Total effect, c	0.800	(0.726, 0.873)	0.038	< 0.001	
Direct effect, c′	0.478	(0.379, 0.577)	0.050	< 0.001	59.75
Indirect effect, a^*^b	0.321	(0.233, 0.409)	0.045	< 0.001	40.13

Furthermore, the direct pathway from social capital to health-promoting behaviors (path c) is quantified with a total effect of 0.800 (95% CI: 0.726–0.873). After accounting for the mediator, the direct effect of social capital on health-promoting behaviors (path “c′”) is reduced to 0.321. Importantly, 40.13% of the total effect of social capital on health behaviors is mediated by positive psychological capital. This suggests that positive psychological capital does not merely enhance health-promoting behaviors independently but also amplifies the impact of social capital on these behaviors (Table 3 and Figure 3).

## 4 Discussion

This cross-sectional study among elderly populations in Chinese nursing homes highlights the significant roles of social capital and positive psychological capital in promoting health behaviors. The findings reveal that social capital directly fosters health behaviors in the elderly and that its impact is enhanced through the mediating role of positive psychological capital, which serves as a crucial bridge between social capital and health-promoting behaviors. Specifically, social capital, particularly through improved community connections and opportunities for participation in community organizations, not only provides psychological benefits but also offers greater access to support and resources, which can supplement formal healthcare services and positively affect the elderly's health and well-being (Ahmadboukani et al., 2023; Chang et al., 2023). Furthermore, positive psychological capital—comprising self-efficacy, hope, resilience, and optimism—demonstrates substantial benefits for psychological health by reducing loneliness and enhancing subjective well-being, which motivates the elderly to engage in health-promoting behaviors such as physical activity, healthy eating, and social interactions (Ahmadboukani et al., 2023). These findings validate the positive correlations among social capital, positive psychological capital, and health behaviors, confirming the pivotal role of these factors in promoting elderly health. This study not only highlights the direct roles of social and psychological capital in health promotion but also underscores their potential implications for public health policy and aged care service practices.

Our study found that the average health-promoting behavior score among elderly residents in Chinese nursing homes was 68.25 ± 10.85, indicating relatively low engagement in health-promoting behaviors. This score is significantly lower than that reported in other studies. For instance, a meta-analysis of 5,639 elderly individuals found that older adults generally engage in moderate health-promoting lifestyle behaviors (Yue et al., 2018). The low levels of health-promoting behaviors in Chinese nursing homes may stem from several factors, including limited institutional resources, a lack of health education, and insufficient personal health awareness, all of which hinder the adoption of proactive health behaviors (Fang et al., 2015; Li and Shi, 2022).

We found that the average social capital score of elderly residents in nursing homes in the Xiangxi region was 46.83 ± 10.26, significantly lower than the 75.41 ± 8.61 reported for elderly residents in Shenyang, Liaoning Province (Li, 2021). This difference likely reflects regional disparities in the development of social capital, influenced by socioeconomic status, healthcare access, and cultural factors. In economically underdeveloped regions like Xiangxi, where healthcare resources and social support are limited, elderly residents in nursing homes may experience greater social isolation, reducing their social capital. Previous studies highlight that social capital varies across different socioeconomic and cultural contexts, emphasizing the need to study it in developing and disadvantaged regions (Cain et al., 2018). In more developed areas, better community resources, economic stability, and social participation opportunities typically foster higher levels of social capital (Cain et al., 2018). Conversely, in regions like Xiangxi, limited resources hinder the formation of strong social networks and community ties, leading to lower individual social capital.

We found that elderly residents in nursing homes in the Xiangxi region scored 72.48 ± 6.39 in positive psychological capital, slightly lower than the 75.77 ± 5.82 reported by Han Jing et al. in Tangshan (Jing et al., 2021) and much lower than the 87.56 ± 8.07 observed in community-dwelling elderly individuals by Shi (2013). This trend suggests that nursing home residents generally exhibit lower psychological capital than their community-dwelling counterparts. Several factors may contribute to this difference, particularly the social and environmental conditions of nursing home life. Compared to community residents, nursing home residents typically have fewer social opportunities, with structured and repetitive activities that lack the stimulation and variety found in community settings (Moyle et al., 2015; Nygaard et al., 2020). This limited social participation may hinder social capital development and reduce psychological resilience, optimism, and overall psychological capital. Additionally, the transition to a nursing home represents a significant life change that can challenge elderly individuals' adaptability and lead to a loss of autonomy, negatively impacting self-efficacy and psychological capital (Yong et al., 2021). Furthermore, while many nursing homes provide basic care, they often lack sufficient psychological and emotional support, exacerbating unmet psychological needs and further lowering psychological capital (Clare et al., 2008; Nygaard et al., 2020).

This study further explored the mediating role of positive psychological capital between social capital and health-promoting behaviors, confirming Research Hypothesis 4. Mediation analysis indicated that the mediating effect of positive psychological capital accounted for 40.13% of the total effect, clearly demonstrating that social capital not only directly influences health-promoting behaviors but also indirectly promotes these behaviors by enhancing positive psychological capital. This finding underscores the necessity of not solely relying on single-dimensional social or psychological interventions when developing health promotion strategies for the elderly. To improve health-promoting behaviors and quality of life for the elderly, a multidimensional strategy is required (Aronson, 2020). Firstly, the strengthening of social capital, including enhancing social support networks, encouraging social participation among the elderly, and fostering a community culture of reciprocity, provides the elderly with necessary external resources and social opportunities, all of which are directly beneficial for the enactment of health-promoting behaviors (Chang et al., 2023). Additionally, social capital indirectly promotes health behaviors in the elderly by influencing positive psychological states such as self-efficacy, hope, resilience, and optimism (Ahmadboukani et al., 2023), such as regular exercise, healthy eating, and active participation in social activities.

Therefore, effective interventions for elderly residents in nursing homes should comprehensively consider the development of both social capital and psychological capital, as their synergistic effects can significantly enhance the health behaviors and quality of life of the elderly. First, nursing homes can regularly organize social activities and health education workshops, which not only provide a platform for social interaction but also enhance residents' awareness of health information and improve their psychological readiness to face health challenges. Through these activities, elderly individuals can strengthen their sense of social support while acquiring more health knowledge, which in turn can help modify unhealthy behaviors. Secondly, nursing homes should offer psychological counseling services and positive psychology training to help elderly residents improve their intrinsic motivation and psychological adaptation. Such interventions can enhance their psychological resilience, enabling them to maintain a positive mindset and adopt effective coping strategies when dealing with health issues. For example, psychological counseling can help alleviate negative emotions such as anxiety and depression, while mindfulness meditation training can improve emotional regulation skills. Additionally, policymakers should encourage nursing homes to integrate psychological health and social capital into resource allocation, ensuring the long-term sustainability of these interventions. The government can implement relevant policies to provide financial support for nursing homes, encouraging them to build social networks based on social capital, while also training caregivers in skills that promote psychological capital. Caregivers should regularly receive training to ensure the effective implementation of these comprehensive interventions.

### 4.1 Research strengths

This study employed a rigorous cluster sampling method, randomly selecting samples from multiple nursing homes, effectively ensuring the representativeness and statistical validity of the results. This approach not only increased the diversity of the sample but also enhanced the general applicability of the research findings, allowing the results to more accurately reflect the actual conditions of a broad elderly population. Additionally, another significant strength of this study lies in the uniqueness of its research focus. We delved into the relationships between social capital, psychological capital, and health behaviors among elderly residents in nursing homes, a relatively underexplored area in existing literature. These results reveal how social capital and psychological capital influence elderly health behaviors through various mechanisms, thus providing a theoretical basis for formulating effective intervention measures. Particularly within the specific social environment of nursing homes, these findings have important practical implications, offering scientific strategies for improving the quality of life and health status of the elderly.

### 4.2 Research limitations

Firstly, due to its cross-sectional design, this study can only highlight correlations, not causations, among social capital, positive psychological capital, and health-promoting behaviors among elderly residents in nursing homes. Longitudinal or intervention studies are needed to establish the directionality and causality of these relationships. Secondly, the study's focus on the underdeveloped Xiangxi region limits the generalizability of the findings to broader elderly populations in other parts of China. The socioeconomic conditions of the Xiangxi region, including lower income levels, limited access to healthcare, and reduced social support, may influence both the social capital and health-promoting behaviors of elderly residents. These regional socioeconomic differences could potentially confound the observed relationships, making it difficult to generalize the results to regions with more developed economic and healthcare systems. Future research should compare different regions with varying socioeconomic conditions to verify these results across more diverse settings and assess how regional factors may shape the relationships between social capital, psychological capital, and health behaviors. Lastly, reliance on self-reported data may introduce biases, such as memory inaccuracies or social desirability effects. Future studies should incorporate objective measurements to validate the findings and reduce potential biases.

### 4.3 Conclusion

This study demonstrates a positive correlation among social capital, positive psychological capital, and health-promoting behaviors in elderly nursing home residents, with positive psychological capital mediating this relationship. For more effective health interventions, it is crucial to consider both social and psychological capitals. Future research should explore these relationships through longitudinal studies to better understand causality and direction. Additionally, comparing these dynamics in nursing home residents with community-dwelling elderly could enhance intervention strategies tailored to different living environments.

## Data Availability

The original contributions presented in the study are included in the article/supplementary material, further inquiries can be directed to the corresponding author.
